# Association Between the Immunohistochemistry Expression of E-cadherin, Beta-Catenin, and CD44 in Colorectal Adenocarcinoma

**DOI:** 10.7759/cureus.35686

**Published:** 2023-03-02

**Authors:** Amrutha Tunuguntla, T.N. Suresh, Sreeramulu PN

**Affiliations:** 1 Department of Pathology, Sri Devaraj Urs Medical College, Kolar, IND; 2 Department of Surgery, R.L. Jalappa Hospital and Research Centre, Sri Devaraj Urs Academy of Higher Education and Research, Kolar, IND

**Keywords:** e-cadherin, beta-catenin, cd44, immunohistochemistry, cancer stem cells, epithelial-mesenchymal transition, colorectal carcinoma

## Abstract

Background

Colorectal cancer is a leading cause of cancer-related deaths worldwide, and epithelial-mesenchymal transition (EMT) plays an important role in cancer metastasis. In EMT, there is downregulation of E-cadherin, an intracellular adhesion molecule, as well as mutations in beta-catenin genes. On immunohistochemistry (IHC), the expression of CD44 portrays stem cell differentiation, which, in turn, is strongly associated with EMT. Thus, newer targeted therapies can be advised based on the expression of EMT and stem cell differentiation.

Aims and objectives

To determine the IHC expression of E-cadherin, beta-catenin, and CD44 in colorectal adenocarcinoma and find the association of the IHC expression of E-cadherin, beta-catenin, and CD44 with the histopathological grade, stage, lymph node metastasis, and lymphovascular invasion of colorectal adenocarcinoma.

Materials and methods

Fifty histologically proven cases of colorectal adenocarcinoma from 2016 to 2021 were included in this study, and clinicopathological data including age, gender, grading, TNM (tumour, node, and metastasis) staging, and lymph node metastasis were collected and hematoxylin and eosin slides were reviewed. IHC staining for E-cadherin, beta-catenin, and CD44 was done for all cases using the peroxidase and anti-peroxidase method, and the results were analysed.

Results

Peak incidence occurred in the 61-70 years age group (36%), and the most common site of the tumour was the rectal area (48%). The majority of the cases were in TNM stage II (37.3%), and a low expression of E-cadherin was found to be associated with higher T stage (p = 0.03), TNM staging (p = 0.04), as well as the presence of lymph node metastasis (p = 0.006). High beta-catenin expression was observed to have a significant correlation with a higher T stage (p = 0.006) and TNM staging (p = 0.005), while high CD44 expression was found to be associated with lymph node metastasis (p = 0.01). Altered expression of EMT-related proteins (E-cadherin and beta-catenin) showed a significant correlation with higher T stage (p = 0.03), TNM staging (p = 0.016), and lymph node metastasis (0.04).

Conclusions

EMT and cancer stem cell IHC markers are biomarkers for aggressive tumour growth and lymph node metastasis. Hence, EMT markers (E-cadherin and beta-catenin) and cancer stem cell markers (CD44) can be used as prognostic markers.

## Introduction

Colorectal carcinoma (CRC) is a serious health problem, and it is responsible for many deaths worldwide [1]. In India, the incidence rate among women is 3.9 per 100,000 population and 4.4 per 100,000 population among men annually [2]. CRC ranks third amongst all cancers afflicting Indian men and women. Both genetic and epigenetic abnormalities lead to a combination of molecular events that lead to colonic adenocarcinoma. The two main genetic pathways are the adenomatous polyposis coli (APC)/beta-catenin pathway and the microsatellite instability (MSI) pathway [1]. The APC protein binds and promotes the degradation of beta-catenin, a component of the Wnt signalling pathway. This also plays a role in promoting epithelial-mesenchymal transition (EMT) [3].

EMT plays an important role in the development of embryos, cancer metastasis, and the process of fibrosis. Activation of EMT during cancer progression empowers cancer cells to acquire the ability to migrate, become invasive, and gain stem-like features [3]. E-cadherin is a transmembrane protein that links the plasma membrane of two cells together. In EMT, there is a downregulation of E-cadherin, which is a strong intracellular adhesion molecule and an autocrine motility factor (AMFR) [4]. Beta-catenin, which is a glycoprotein, is a central component of adhesion junctions that has the ability to bind to the E-cadherin in epithelial cells, thereby stabilising the cytoskeleton of cells and preventing any abnormal cell growth [5]. Mutations in beta-catenin cause its accumulation, which eventually leads to classic adenoma-carcinoma sequences [6].

EMT activation has been found to lead to the generation of cancer stem cells (CSCs). In transformed epithelial cells, EMT induction culminates by enhancing cells with stem-like traits, which, in turn, are responsible for primary tumour initiation and its accelerated metastasis [7]. In CRC, stem cell differentiation occurs by the expression of CD44, which is significantly associated with EMT and represents the target treatment [8]. However, available literature contains only a few studies pertaining to these markers among the Indian population. Therefore, this study aims to investigate the association between the immunohistochemistry (IHC) expression of E-cadherin, beta-catenin, and CD44 with the histopathological grade, stage, lymph node metastasis, and lymphovascular invasion of CRC. The results of this study could help in decision-making for newer targeted therapies for CRC.

## Materials and methods

This is a laboratory-based observational study. The Institutional Ethics Committee's clearance was taken prior to the study. All surgically resected CRC from 2016 to 2021 were included in the study. Paraffin blocks and slides were retrieved from the department of pathology, while clinical information for histopathological diagnosis was obtained from medical records and pathology reports. All the hematoxylin and eosin slides were screened for histological parameters like tumour grade, stage, lymph node metastasis, and lymphovascular invasion, and IHC was performed for all the cases with EMT markers (E-cadherin and beta-catenin) and CD44 cancer stem cell marker. IHC was performed on all tumour sections using peroxidase and anti-peroxidase methods. Primary prediluted rabbit monoclonal antibodies, E-cadherin (EP6), beta-catenin (EP35), and CD44 (SP37) (PathnSitu, Secunderabad, India) were used.

Grading of IHC

For the interpretation of the immunoreactivity, five representative fields from the tumour were chosen for evaluating the intensity and fraction percentage of positive cells by two independent pathologists.

The staining of E-cadherin and beta-catenin was scored according to the proportion and intensity categories proposed by Allred et al. [9]. The staining intensity was estimated in a four-step scale (0 = none, 1 = weak, 2 = moderate, and 3 = strong), while the fraction of the stained cells was scored according to the following criteria: score 0 = <10%, score 1-11 = 33%, score 2-33 = 66%, and score 3 = >67% positive cancer cells.

The final staining score was assigned based on the multiplication of the staining intensity as well as the percentage of the positive cells and graded as follows: 0 = 0, 1 = 1-3, 2 = 4-6, and 3 = 7-9; low expression = 0 or 1 and high expression = 2 or 3 [9,10].

The staining of CD44 was scored according to the proportion and intensity categories proposed by Fang et al. [11]. The staining intensity was estimated in a four-step scale (0 = none, 1 = weak, 2 = moderate, and 3 = strong), while the fraction of the stained cells was scored according to the following criteria: score 0 = <10%, score 1 = 11-50%, score 2 = 51-80%, and score 3 = >80% [11].

The final staining score was assigned based on the multiplication of the staining intensity as well as the percentage of the positive cells and graded as follows: 0 = 0, 1 = 1-3, 2 = 4-6, and 3 = 7-9; low expression = 0 or 1 and high expression = 2 or 3 [11].

Epithelial-Mesenchymal Transition

All the cases showing altered protein expression of EMT markers were regarded as positive for EMT [12,13]. In the current study, low expression of E-cadherin and high expression of beta-catenin together were taken as positive for EMT.

Statistical analysis

The data entry was done using Microsoft Excel (Microsoft Corporation, Redmond, WA) and statistically analysed using Statistical Package for Social Sciences (SPSS version 22, IBM Corp., Armonk, NY). Categorical variables were summarised using frequencies and proportions, and all results were presented in tabular form. The groups were tested for statistical significance using the chi-square test and Fisher's exact test, and a p-value less than 0.05 was considered statistically significant.

## Results

A total of 50 CRC cases were taken in this study. The peak incidence was in the 61-70 years age group (36%). Most patients were females (52%) and 48% were males, with a male-to-female ratio of 0.92:1. The most common tumour site was the colon (52%), including ascending colon, transverse colon, descending colon, and sigmoid colon, and 48% were in the rectum.

Out of the 50 cases, the majority had moderately differentiated adenocarcinoma (48%), followed by well-differentiated adenocarcinoma (36%), poorly differentiated adenocarcinoma (10%), and mucinous adenocarcinoma (6%). We grouped all the cases of well-differentiated adenocarcinoma, moderately differentiated adenocarcinoma, and mucinous adenocarcinoma as low grade, while poorly differentiated adenocarcinoma was considered high grade. Most of the patients (90%) were low grade, while high grade was observed in a few patients (10%).

The majority of the patients in the current study had a pathological T staging of T3 (62%), followed by T2 (26%) and T4 (12%). Also, the majority were in stage II (40%), followed by stage III (34%), stage I (24%), and stage IV (2%). Among the study participants, lymph node metastasis was present in 38%, while lymphovascular invasion was present in only 6%.

**Figure 1:**
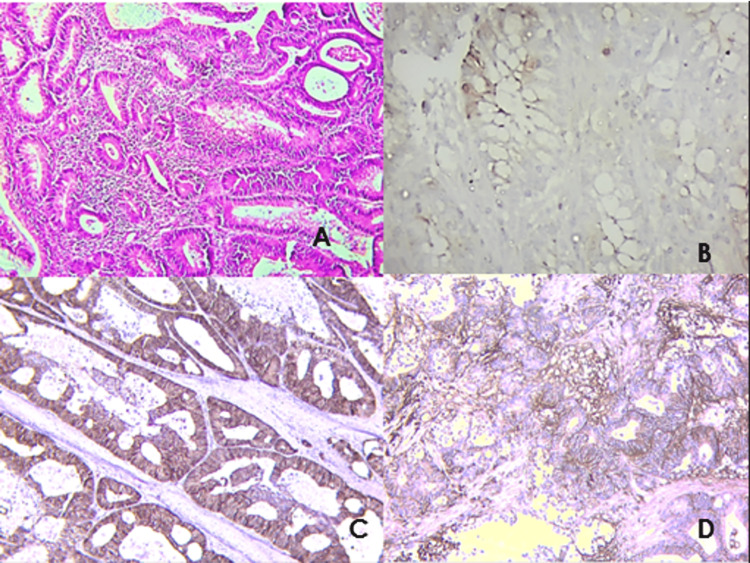
(A) Hematoxylin and eosin stained section showing well-differentiated adenocarcinoma (x100). (B) Microphotograph of E-cadherin immunohistochemistry (IHC) staining showing low expression with intensity = 2 and % fraction = 1 (x400). (C) Microphotograph of beta-catenin IHC staining showing high expression with intensity = 3 and % fraction = 3 (x100). (D) Microphotograph of beta-catenin IHC staining showing high expression with intensity = 3 and % fraction = 3 (x100).

The majority of the patients (60%) had low expression of E-cadherin levels, while 40% had high expression. Also, most participants (60%) had high expression of beta-catenin levels, while 40% had low expression of beta-catenin. CD44 expression was high in 58%, while 42% had low expression (Figure 1).

With respect to the E-cadherin, low expression was found to be significantly associated with higher T stage (p = 0.03), TNM (tumour, node, and metastasis) staging (p = 0.04), and the presence of lymph node metastasis (p = 0.006). High beta-catenin expression was observed to have a significant correlation with higher T stage (p = 0.006) and TNM staging (p = 0.005), while high CD44 expression was found to be significantly associated with lymph node metastasis (p = 0.01) (Table 1).

**Table 1:** Expression of E-cadherin, beta-catenin, and CD44 with respect to grading, staging, lymph node metastasis, and lymphovascular invasion. LN Mets = lymph node metastasis; LVI = lymphovascular invasion.

		E-cadherin			Beta-catenin			CD44			
Parameters		High expression	Low expression	P-value	High expression	Low expression	P-value	High expression	Low expression	P-value	
Grade	High	1	4	0.33	3	2	1	3	3	0.7	
	Low	19	26		27	18		26	18		
T stage	T2	9	4	0.03	2	11	0.00	4	9	0.06	
	T3	10	21		23	8		21	10		
	T4	1	5		5	1		4	2		
TNM staging	I	9	3	0.04	2	10	0.005	3	9	0.064	
	II	9	10		13	6		11	8		
	III	2	16		14	4		14	4		
	IV	0	1		1	0		1	0		
LN Mets	Present	3	16	0.006	14	5	0.12	15	4	0.018	
	Absent	17	14		16	15		14	17		
LVI	Present	2	1	0.30	2	1	0.81	3	0	0.25	
	Absent	18	29		28	19		26	21		

Altered expression of EMT-related proteins (E-cadherin and beta-catenin) showed a significant correlation with higher T stage (p = 0.03), TNM staging (p = 0.016), and lymph node metastasis (p = 0.04).

## Discussion

CRC is one of the most common malignant tumours globally, ranking third in incidence and fourth in mortality, and approximately accounting for 10% of the global cancer burden [12]. Conventional prognostic parameters for CRC include TNM staging, tumour grade, lymphatic, perineural invasion, venous invasion, and tumour border architecture [13-16]. The activation of EMT during cancer growth enables cancer cells to develop characteristics, including migratory, invasive, and stem-like traits [3].

In the present study, the mean age was 68 years, which was comparable to findings from similar studies [17-19]. In the current study, 52% were of the female gender, while 48% were male. Conversely, the studies by Iseki et al., Gomaa et al., and Melincovici et al. had a slight male predominance [18-20]. In the study by Choi et al. [17], slight female predominance was noted, which is similar to the findings from the present study. Archilla et al. reported the rectum was a more common tumour site than the colon [21]. Whereas other studies by Rajkumar et al. and Iseki et al. reported a higher incidence in the colon, which was similar to our study [19,22].

In the present study, 62% of cases were categorised in the T3 stage, and comparable findings were noted by Gomaa et al. and Melincovici et al. [18,20], whereas the T4 stage was the predominant category in the study by Iseki et al. [19]. In our study, most cases were stage II cancers (40%), similar to reports from the study by Banias et al. [23]. On the contrary, studies by Melincovici et al. and Choi et al. had a higher number of stage III and stage IV cancers [17,18].

In the current study, most participants (60%) had low E-cadherin expression, while 40% had high expression of E-cadherin. Choi et al. and Melincovici et al. found a substantial association between the loss of expression of E-cadherin and cancer grading [17,18]; however, no such association was found in our study. Nevertheless, T stage, lymph nodal metastasis, and TNM staging showed a significant association with loss of expression of E-cadherin in our study, which was relatable to the study done by Choi et al. [17]. The lymphovascular invasion did not show any significant association with the loss of E-cadherin expression. According to Yun et al., low E-cadherin expression is correlated to poor survival outcomes [24]. Gomaa et al. found that some prognostic parameters are associated with decreased E-cadherin expression in CRC, which is also associated with disease relapse in primary CRC and independent predictor for relapse of the disease [20]. For CRC development, these results could indicate that the Wnt/beta-catenin pathway is crucial (Table 2).

**Table 2:** Comparison of the statistical analysis of loss of E-cadherin expression to various histopathological parameters with other studies. T staging = tumour staging; LN Mets = lymph node metastasis; TNM = tumour, node, and metastasis.

Loss of E-cadherin	Choi et al. [17] (2017) (n = 286)	Melincovici et al. [18] (2020) (n = 31)	Iseki et al. [19] (2017) (n = 49)	Gomaa et al. [20] (2021) (n = 196)	Present study (n = 50)
	P-value	P-value	P-value	P-value	P-value
Grading	0.00	0.018	0.81	0.46	0.336
T staging	0.04	Not specified	0.44	0.7	0.033
LN Mets	0.001	0.28	0.24	0.8	0.006
TNM staging	0.001	0.81	Not specified	Not specified	0.04
Lymphovascular invasion	0.009	0.39	0.3	0.12	0.30

Increased expression of beta-catenin showed a strong association with TNM staging, which was also seen in the study by Choi et al. [17]. The rest of the parameters did not show significant p-value as noted in other studies such as that by Gomaa et al. [20] and Melincovici et al. [18] (Table 3).

**Table 3:** Comparison of statistical analysis of the accumulation of beta-catenin expression to various histopathological parameters with other studies. T staging = tumour staging; LN Mets = lymph node metastasis; TNM = tumour, node, and metastasis.

High expression of beta-catenin	Gomaa et al. [20] (2021) (n = 196)	Melincovici et al. [18] (2020) (n = 31)	Choi et al. [17] (2017) (n = 286)	Present study (n = 50)
	P-value	P-value	P-value	P-value
Grading	0.71	0.23	0.001	1
T staging	0.39	Not specified	0.59	0.000
LN Mets	0.95	0.046	0.012	0.122
TNM staging	Not specified	0.93	0.034	0.005
Lymphovascular invasion	0.41	0.7	0.007	0.80

In the studies by Bhattacharya et al. [6] and Wong et al. [25], a major statistically positive correlation was achieved between beta-catenin subcellular localisation and their corresponding membranous, cytoplasmic, and nuclear score related to the American Joint Committee on Cancer (AJCC)-TNM stage (r = 0.512; p < 0.001) of colorectal adenocarcinoma. However, the study by Gomaa et al. [20] stated that loss of beta-catenin expression was significantly associated with aggressive behaviour, high stage, and distant metastases, and aggressive CRC is associated with a decreased expression of beta-catenin.

CD44, a transmembrane glycoprotein of class 1, is essential for lymphocyte homing, angiogenesis, inflammation, cell proliferation, and motility, and in conjunction with hyaluronic acid and glycosaminoglycans, it plays a key role in cell-to-extracellular matrix adhesion [17]. According to the results of the meta-analysis, CD44 overexpression in CRC is an adverse prognostic marker that predicts a high grade and metastases in lymph nodes and distant regions [8].

In the present study, the high expression of CD44 indicated a significant association with lymph nodal metastasis, and related results were also noted in the studies by Wang et al. [26] and Choi et al. [17]. The rest of the parameters did not show any statistical significance.

The meta-analysis by Wang et al. concluded that poor differentiation, lymph node metastases, distant metastasis, and poor overall survival were linked to high CD44 expression [26]. Iseki et al. reported that CD44 expression, which is a poor prognosis indicator for patients undergoing curative surgery, was not shown to be a predictive predictor for patients with unresectable metastatic CRC (Table 4) [19].

**Table 4:** Comparison of the statistical analysis of CD44 expression to various histopathological parameters with other studies T staging = tumour staging; LN Mets = lymph node metastasis; TNM = tumour, node, and metastasis.

High expression of CD44	Iseki et al. [19] (2017) (n = 49)	Choi et al. [17] (2017) (n = 286)	Wang et al. [26] (2022) (48 studies meta-analysis)	Present study (n = 50)
	P-value	P-value	P-value	P-value
Grading	0.55	0.36	0.25	0.7
T staging	0.07	0.02	0.46	0.068
LN Mets	0.71	0.005	0.04	0.018
TNM staging	Not specified	Not specified	Not specified	0.064
Lymphovascular invasion	0.18	0.51	0.153	0.25

There is a strong relationship between CD44 and EMT in CRC, which is defined by the loss of epithelial markers such as E-cadherin and the acquisition of mesenchymal features in tumour cells. EMT results in the improvement of CD44-positive cancer stem cells [8], and in certain studies, EMT has been found to suppress the growth of stem cell-like features [27,28], which opposes the idea of EMT-induced stemness.

Similar results were seen in our study, where altered protein expression of EMT (E-cadherin and beta-catenin) did not show any significant association with CD44. Also, similar findings were reported in the studies by Choi et al. [17] and Ngan et al. [29].

Cells undergo EMT to become mesenchymal, and epithelial cells lose cell-to-cell contact and cell polarity during EMT, thus increasing their mobility and invasiveness. EMT involves the downregulation of epithelial markers and abnormal overexpression of mesenchymal markers [8].

Our results were similar to those from the studies by Choi et al. [17] and Ngan et al. [29]. Also, previous studies observed that EMT-related marker expression is linked to tumour size, differentiation, growth patterns, metastasis, and poor prognosis [22]. Ribatti et al. concluded that EMT controls tumour development, progression, and metastasis in the tumour microenvironment [3]. Hence, these EMT markers (E-cadherin and beta-catenin) and cancer stem cell marker (CD44) can be used as prognostic markers for predicting tumour growth and lymph node metastasis (Table 5).

**Table 5:** Comparison of statistical analysis of altered protein expression of EMT to various histopathological parameters with other studies EMT: epithelial-mesenchymal transition; T staging = tumour staging; LN Mets = lymph node metastasis; TNM = tumour, node, and metastasis.

Altered protein expression of EMT markers	Choi et al. [17] (2017) (n = 286)	Ngan et al. [29] (2017) (n = 140)	Present study (n = 50)
	P-value	P-value	P-value
Grading	0.007	0.643	0.57
T staging	0.7	0.54	0.032
LN Mets	0.026	0.03	0.04
TNM staging	0.05	-	0.016

Limitations

This is a unicentric study and the sample size is small, which may affect the results as only 50 patient samples were available during the study period and the follow-up could not be done for all these patients.

## Conclusions

In this study, altered expressions of EMT-related proteins showed a significant association with TNM staging and lymph node metastasis in CRC. A high expression of CD44 was significantly associated with lymph node metastasis. EMT and cancer stem cell IHC markers are possible biomarkers for aggressive tumour behaviour and lymph node metastasis.
